# Sperm Morphology and Male Age in Black-Throated Blue Warblers, an Ecological Model System

**DOI:** 10.3390/ani10071175

**Published:** 2020-07-10

**Authors:** Emily Cramer, Nicole Krauss, Tricia Rowlison, Pierre Comizzoli

**Affiliations:** 1 Smithsonian Conservation Biology Institute, National Zoological Park, Washington DC, 20008, USA; RowlisonT@si.edu (T.R.); comizzolip@si.edu (P.C.); 2 Cornell Lab of Ornithology, Cornell University, Ithaca, NY 14850, USA; 3 Premedical Education Department, Weill Cornell Medicine-Qatar, Doha 24144, Qatar; 4 School of Biological Sciences, Washington State University, Pullman, WA 99164, USA; kraussne@gmail.com

**Keywords:** age effects, birds, extra-pair paternity, Parulidae, post-copulatory sexual selection, sperm morphology, spermatozoa, wildlife

## Abstract

**Simple Summary:**

Sperm cell characteristics can impact the number of offspring a male sires, particularly when females copulate with more than one male in a reproductive cycle. Females copulate with multiple males in many songbird species, but for many of these species, little is known about sperm characteristics. In this paper, we examine the shape of sperm cells in black-throated blue warblers, a species where multiple copulations are common but where sperm morphology was previously unknown. Sperm shape did not correlate with plumage characteristics that may make males more successful at gaining copulation partners. However, older males tended to have longer sperm cells. Previous work shows that older males sire more offspring, and work in other species indicates that longer sperm cells are more effective at fertilizing eggs. Thus, sperm shape may help explain the higher success of older males in this species.

**Abstract:**

Extra-pair paternity may drive selection on spermatozoa and ejaculate characteristics through sperm competition and cryptic female choice. Here, we examine sperm morphology in the black-throated blue warbler (*Setophaga caerulescens*), an ecological model species where extra-pair paternity is frequent and is linked with male age. We test whether sperm morphology relates to several aspects of male phenotype known or suspected to affect extra-pair paternity success. Sperm morphology did not correlate with the size of the white wing spot, a social status signal, nor with the volume of the cloacal protuberance. However, older males tended to have longer sperm cells. Although the sample size was limited, this pattern is intriguing, as longer cells may be advantageous in post-copulatory sexual selection and older males have larger testes and higher extra-pair paternity success in this species. Changes in sperm morphology with age are not observed in other birds, though they have been observed in insects and fishes. More research on sperm morphology is needed to clarify its role in extra-pair fertilizations in this well-studied species.

## 1. Introduction

Sperm cells evolve in response to diverse selection pressures [1]. Among these pressures are sperm competition and cryptic female choice [2,3], which can act when a female copulates with more than one male in a reproductive cycle, for instance in polyandrous mating systems or socially monogamous systems with extra-pair paternity (i.e., paternity by males other than the female’s social partner). Consistent with increased selection due to sperm competition and cryptic female choice, passerine bird species with higher levels of extra-pair paternity exhibit reduced among-male variation in sperm total length [4,5,6], longer spermatozoa [7,8], and faster change in sperm length over evolutionary time [9]. Sperm competition experiments with captive zebra finches (*Taeniopygia guttata*) show that males with longer and faster-swimming spermatozoa have higher fertilization success [10]. Despite the potential importance of sperm and ejaculate traits in extra-pair paternity, few studies have investigated sperm biology within passerine species. 

Black-throated blue warblers (*Setophaga carulescens*) are socially monogamous, open-cup nesting songbirds with substantial extra-pair paternity (approximately 43% of offspring in 55% of broods), typically occurring among neighboring territories [11,12]. Males with lower food abundance on their territories are more likely to sire extra-pair paternity, and to sire offspring at a greater distance from their home territories, suggesting a possible role of foraging forays in extra-pair mating [11,13]. Older males are also more likely to sire extra-pair offspring [11]. Males and females differ substantially in plumage, with females being drab olive colored and males having a blue back, black face and flanks, and white underside [14]. Both sexes have a white wing patch; it is larger in males [14] and affects male-male interactions, particularly for young males [15]. Being sexually dimorphic, dorsal coloration and wing patch size may be hypothesized to affect extra-pair copulations, although one study found no relationship between wing patch and parentage [16]. Despite being the focus of intense study over many years [17], and particularly in the context of extra-pair paternity [11,12,13,18,19,20], sperm morphology was previously undescribed in this species.

Here, we examine the morphology of spermatozoa in black-throated blue warblers and assess its relationship to male traits known or hypothesized to affect extra-pair paternity: Male age, plumage, and cloacal protuberance volume (which may be used as a proxy for sperm production [4]). We focus on total sperm length, within-male variation in total sperm length, and the relative flagellum length, since sperm with longer flagella relative to head may swim more rapidly and thus have higher chances of fertilizing the eggs [7,21,22]; but see [8,23]. Because the direction and strength of correlation between male and ejaculate traits varies substantially in other taxa [24,25,26], no specific predictions were made. We show that older males tended to have longer sperm cells, an intriguing pattern given the importance of male age in extra-pair paternity in this species. 

## 2. Materials and Methods 

### 2.1. Animals and Sampling Procedures

Wild, free-living males were captured by mistnetting, from 22 May 2016 to 28 June 2016 at the Hubbard Brook Experimental Forest, Woodstock, New Hampshire, USA (43°56′ N, 71°45′ W). Birds were banded, bled, and measured for inclusion in on-going long-term studies. When weather and bird condition permitted slightly longer safe handling time, we collected a single semen sample per male by cloacal massage (*n* = 13 males). Semen samples were mixed with approximately 20 μL phosphate-buffered saline, and we transferred the sample into 5% formaldehyde immediately. We measured (1) the size of the white wing patch using digital calipers to the nearest 0.1 mm, placed around the longest extent of white visible while the wing was closed (*n* = 12; [15]), (2) the percentage of black feathers in the male’s backs, which was estimated while the bander held the bird in a modified bander’s grip to expose the back (*n* = 11 males [27]); (3) and the length and diameter of the cloacal protuberance (*n* = 10 males). Cloacal protuberance volume was calculated as length times radius squared times pi. Age was assigned as second-year (SY; first breeding season) if the alula had green edging or after second year (ASY) if the alula was pure blue [27]. Only four males had nests that survived until genetic sampling of offspring, so we did not attempt to examine genetic parentage. Male measurements are in Table S1.

### 2.2. Sperm Morphology Measurements

**Figure 1:**
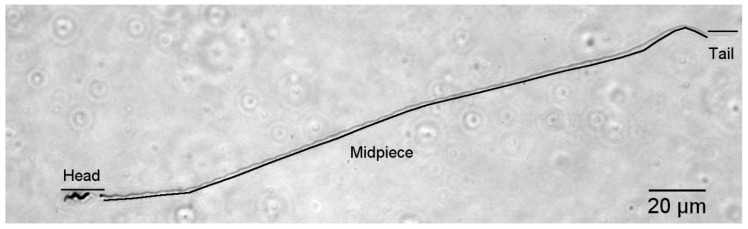
A single, Coomassie-blue stained spermatozoan from a black-throated blue warbler (400× total magnification). The sections measured as head, midpiece and tail (exposed flagellum) are labelled. The unstained portion of the head fluoresced strongly with a DAPI stain in pilot work.

For each male, approximately 10 μL fixed semen sample was streaked onto a microscope slide, where it was allowed to air-dry before being immersed in Coomassie Blue stain for 1.5 min. Excess stain was rinsed off, a cover slip was applied using Permount, and slides were examined at 400× total magnification with an Olympus BX 41 microscope (Olympus, NewYork, NY, USA; the highest magnification allowing entire sperm cells in one field of view). Twenty morphologically normal sperm cells were photographed (Spot Imaging software, Sterling Heights, MI, USA), and images were analyzed using Image J software (National Institutes of Health, Bethesda, MD, USA) [28]. Cells were considered morphologically normal (e.g., Figure 1) if they exhibited a generally helical superstructure from the acrosome to the end of the midpiece and if there was no evidence of tail breakage. One SY male was excluded because of apparent damage to sperm cells, with the mitochondrial helix often appearing unwound. We measured the length of the head (including acrosome), midpiece, and tail (exposed flagellum; Figure 1), then calculated total sperm length as the sum of these components [8]. Raw data are in Table S2.

Flagellum length was the sum of midpiece and exposed flagellum. The coefficient of variation in sperm length within males (CV_wm_) was calculated as standard deviation in total length, divided by mean total length [29]. Because relatively few males were sampled, we applied a correction factor to avoid bias due to small sample sizes in calculating the coefficient of variation among males (CV_am_ = (standard deviation/mean) × (1 + 1/(4n)) [4,29]. 

All measurements were taken by one person and were blind with respect to male phenotype. Measurements were repeatable (repeatability ± SE as assessed using package rptR [30] on four haphazardly chosen sperm cells from each of five males, with all 20 cells measured five times on different days; head 0.59 ± 0.10; midpiece 0.94 ± 0.02; tail 0.80 ± 0.06; total sperm length 0.99 ± 0.01; all *p* < 0.05 by likelihood ratio test; data in Table S3).

### 2.3. Statistical Analysis

To test whether males differed in sperm morphology, we performed ANOVA using data from individual sperm cells. To examine relationships among sperm component lengths, and between sperm measures and male phenotypes, we used the average length of each sperm component or measurement for each male. To test for correlations among different sperm components lengths, we performed linear regression among all pairs of sperm components (head, midpiece, and tail). To test whether sperm measurements (total sperm length, CV_wm_, and the relative length of the flagellum) related to sexual phenotypes (male age, the size of the wing spot, the percent of black feathers on the back, and the volume of the cloacal protuberance), we again used linear regression. For tests on relative flagellum length, we used a linear regression with flagellum length as the response variable, head length as a covariate, and one male phenotypic trait per model as the predictor of interest. For all other sperm measurements, we included only the male phenotypic trait as a predictor. Results were similar using data on individual sperm cells as the response variables, in mixed models controlling for male identity as a random effect (not shown; reporting only mean-level models was preferred for simplicity, since the single-cell approach is not available for CV_wm_). We further tested whether total sperm length changed with date of capture (e.g., [31] using linear regression on mean total sperm length). 

To correct for multiple testing, we used false discovery rate correction, performing separate corrections for (1) comparisons of mean lengths across males; (2) assessing relationships among sperm component lengths; and (3) assessing relationships between sperm measurements and male phenotype. Uncorrected *p*-values are shown except where noted otherwise. All analyses were conducted in R version.3.6.1 (R Development Core Team 2019, Vienna, Austria [32]). Code used in analysis are in File S1.

### 2.4. Ethics Statement

This research was conducted with approval from the National Zoological Park Animal Care and Use Committee (14–20). The federal banding permit numbers is 23885. 

## 3. Results

Total sperm length (mean ± SD) was 247.66 ± 5.11 μm and CV_am_ was 2.1% (Table 1). Among-male variation was statistically significant for head, midpiece, and total sperm length, and it approached significance for tail (Table 1; *p*-values not qualitatively changed by FDR correction). 

**Figure 2:**
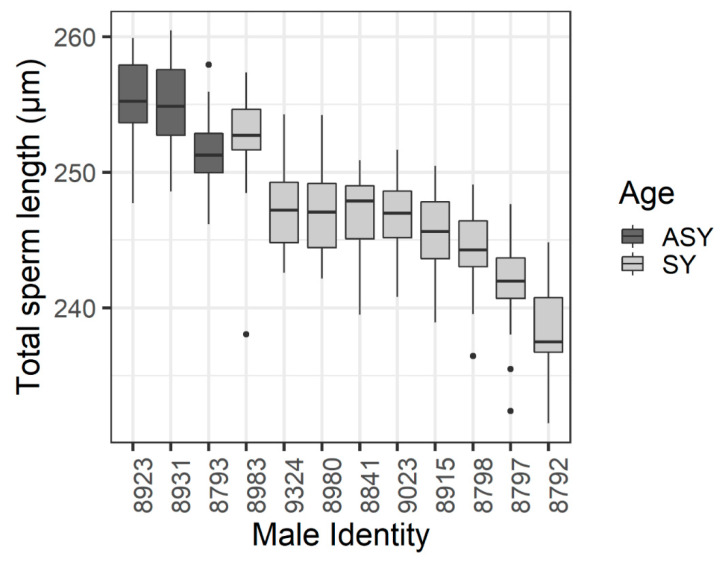
Sperm length from males in different age categories. Older males (in their second or later breeding season, ASY) are in dark grey, and young males in their first-breeding season (SY) are in light grey. Males are in order by total length within an age category. Boxplots show median, interquartile range (IQR), and greatest data extent within 1.5 × IQR, with more extreme data points indicated by dots. *n* = 20 cells per male.

There were no significant correlations among sperm component lengths (head vs. midpiece, F_1,10_ = 1.23, *p* = 0.29; head vs. tail, F_1,10_ = 0.144, *p* = 0.71, midpiece vs. tail F_1,10_ = 2.52, *p* = 0.14). Sperm measurements did not correlate with capture date (total sperm length F_1,10_ = 2.99, *p* = 0.11; flagellum length controlling for head F_1,9_ = 2.81, *p* = 0.13; CV_wm_ F_1,10_ = 0.17, *p* = 0.69). Older males tended to have longer sperm than young males (F_1,10_ = 11.74, uncorrected *p* = 0.006, corrected *p* = 0.08, estimated difference 8.3 ± 2.4 μm; Figure 2), with an accompanying trend for older males to have a longer flagellum relative to head length (F_1,9_ = 9.10, uncorrected *p* = 0.01, corrected *p* = 0.09). No other relationships between sperm traits and male phenotypes approached significance (Table 2). 

## 4. Discussion

Older male black-throated blue warblers tended to have longer sperm, a pattern which has been observed in rove beetles (*Aleochara bilineata* [33]) and guppies (*Poecilia reticulata* [34]). Longer sperm are generally thought to have a competitive advantage over shorter sperm [10,35]. Increasing male age also correlates with greater total testicular volume and more asymmetrical testes in black-throated blue warblers [19], and with larger testes in other bird species ([36] and references therein). Larger testes are expected to allow older males to produce more sperm and therefore increase their chances of success in post-copulatory sexual selection [37]. Indeed, older males are more likely to sire extra-pair offspring in black-throated blue warblers [11] and in many other birds [38]. Recent experimental work in the house sparrow (*Passer domesticus*) suggests that the extra-pair success of older males may be due more to post-copulatory than pre-copulatory processes [39]. While the difference in total sperm length between age groups should be treated as preliminary due to our low sample size, it seems possible that age-dependent changes in reproductive traits such as sperm size and testes size and asymmetry play a role in differential siring success in this system.

In the other passerine birds that have been tested, sperm morphology does not differ between young and old males [40,41,42,43,44]. Furthermore, repeated samples across years of the same individuals show high repeatability in total sperm length, suggesting that between-season changes in sperm morphology within an individual are limited [23,31,42,45,46]. Small within-season changes in morphology, in contrast, appear to be relatively common, occurring in most of the species studied [31,42,43,46] (but see [45]). Older male black-throated blue warblers are more likely to obtain and defend territories with high quality nesting and foraging habitat [47], suggesting that, if they therefore have higher body condition (not assessed in this study), body condition could mediate the greater sperm length observed in older birds. Further study on black-throated blue warblers, with larger sample sizes, is warranted. 

The degree of among-male variation in sperm total length is somewhat higher than expectations based on the extrapair paternity rate of the species (measured CV_am_ 2.1; value of CV_am_ associated with 43% extra-pair young, 1.43, using the calculator provided in [48]). Age-related changes in sperm total length may be partly responsible for this higher-than-expected between-male variation (though note that there is substantial unexplained variation in the relationship, such that our observed value is not extreme [4,48]). Average total sperm length for black-throated blue warblers is similar to other wood-warblers (average for black-throated blue warblers, 247.64 ± 5.11 μm; average for other wood-warblers in [4], 222.78 ± 41.08 μm; average for oscine passerines in [4], 147.82 ± 71.9 μm). 

## 5. Conclusions

Extra-pair paternity is intricately linked with male age, ecology, and time-budget trade-offs in black-throated blue warblers [11,18]. Here we show that age may also affect sperm morphology. Further work is therefore needed to fully how these factors also interact with sperm and ejaculate traits in driving sexual selection in this ecological model system. 

## Figures and Tables

**Table 1:** Sperm component length comparisons for 12 black-throated blue warblers.

Description	Head	Midpiece	Tail	Total Sperm Length (TSL)	CV_am_^1^
Mean (±SD, μm)	14.31 ± 0.44	224.64 ± 5.40	8.71 ± 1.30	247.66 ± 5.11	2.1
ANOVA comparison among males	F_11,228_ = 6.38, *p* < 0.001	F_11,228_ = 19.41, *p* < 0.001	F_11,228_ = 1.75, *p* = 0.06	F_11,228_ = 48.42, *p* < 0.001	n/a

^1^ CV_am_ is the adjusted coefficient of variation of mean sperm length among males. No statistical comparison was performed on this measure.

**Table 2:** Statistical results of the relationship between sperm and ejaculate measurements compared to male traits possibly linked to extra-pair paternity. Uncorrected *p*-values are shown; after correction, no tests had *p* < 0.05.

Predictor	Response Variable		
	Total Sperm Length	Flagellum	CV_wm_^1^
Male age	F_1,10_ = 11.74, *p* = 0.006	F_1,9_ = 9.10, *p* = 0.01	F_1,10_ = 0.31, *p* = 0.59
Size of white wing patch	F_1,10_ = 0.11, *p* = 0.75	F_1,9_ = 0.01, *p* = 0.92	F_1,10_ = 1.19, *p* = 0.30
Percent black dorsal feathers	F_1,9_ = 1.54, *p* = 0.25	F_1,8_ = 1.63, *p* = 0.24	F_1,9_ = 0.10, *p* = 0.76
Volume of cloacal protuberance	F_1,8_ = 0.45, *p* = 0.52	F_1,7_ = 0.19, *p* = 0.68	F_1,8_ = 0.30, *p* = 0.60

^1^ Results were similar when we instead analyzed CV_wm_ using standard deviation in total sperm length as the response variable, with mean total sperm length as a covariate.

## Supplementary Materials

The following are available online at https://www.mdpi.com/2076-2615/10/7/1175/s1, Table S1: Raw data on male characteristics, Table S2: Raw data on sperm morphology used in main analyses, Table S3: Data on sperm morphology used for assessing repeatability, File S1: R code used in analysis.
